# Validation of the 7-item knee replacement patient education questionnaire (KR-PEQ-7), based on the 16-item knee osteoarthritis patient education questionnaire (KOPEQ)

**DOI:** 10.1186/s12891-020-03476-y

**Published:** 2020-07-16

**Authors:** Erika O. Huber, Axel Boger, André Meichtry, Caroline H. Bastiaenen

**Affiliations:** 1 Institute of Physiotherapy, School of Health Professions, Zurich University of Applied Sciences, Technikumstrasse 71, Postfach, 8401 Winterthur, Switzerland; 2 Institute of Physiotherapy, Cantonal Hospital Winterthur, Brauerstrasse 15, 8400 Winterthur, Switzerland; 3 Department of Epidemiology, Research line Functioning, Participation & Rehabilitation CAPHRI School of Public Health and Primary Care, Maastricht University, Maastricht, The Netherlands

**Keywords:** Knee replacement, Knee osteoarthritis, Patient education, Educational intervention, Validity

## Abstract

**Background:**

The aim of this study was to investigate the content validity including item reduction, construct validity and internal consistency of the existing 16-item Knee Osteoarthritis Patient Education Questionnaire. Former research had indicated that a reduction of items was necessary. Participants were patients with severe knee osteoarthritis who, prior to undergoing a knee replacement operation, participated routinely in a preoperative educational intervention.

**Methods:**

A mixed method design was used. The first step was directed at the reduction in the number of items on the 16-item Knee Osteoarthritis Patient Education Questionnaire. Based on a priori hypotheses, this was followed by a cross-sectional validation study, performed to compare the resulting 7-item Knee Replacement Patient Education Questionnaire to a patient-testing Interview Protocol that was tailored to the same patient educational material. Additionally, the revised questionnaire was correlated with both the Short Test of Functional Health Literacy and the Mini-Mental State Examination score.

**Results:**

A relatively high internal consistency was found for the 7-item Knee Replacement Patient Education Questionnaire, with a Cronbach’s alpha of 0.84 (SE: 0.036). Explanatory factor analysis showed no evidence against a one-factor model, with the first and second eigenvalues being 3.8 and 0.31, respectively. Bayesian Estimation of the correlation between the 7-item Knee Replacement Patient Education Questionnaire and the Interview Protocol was 0.78 (mode) (95% HPD 0.58–0.89).

**Conclusions:**

The 7-item Knee Replacement Patient Education Questionnaire shows good psychometric properties and could provide valuable support to health professionals. It can provide valid feedback on how patients waiting for a knee replacement operation experience an applied patient education intervention. Further investigation is needed to assess the applicability of the 7-item Knee Replacement Patient Education Questionnaire to larger samples in different hospitals and countries.

## Background

Patient-reported outcome measures (PROMs) and performance-based tests (PBTs) have been extensively used in research and commonly used in clinical practice to assess treatment outcomes for a variety of musculoskeletal conditions. PROMs provide clinicians with insights into the patient’s perception of his own condition. Contrarily, PBTs measure the clinician’s assessment of the impairments of the patient’s body function [1]. The COSMIN (COnsensus-based Standards for the selection of health Measurement INstruments) team reached international consensus on the taxonomy, terminology, and definitions of measurement properties for health-related PROM [2]. Various studies have synthesized the evidence on the measurement properties of PROM, as well as PBT, in a population with knee and lower limb problems [3–7].

Previous research has addressed the benefits of preoperative education on postoperative outcomes for patients with knee osteoarthritis (OA) on a waiting list for knee replacement (KR) [8]. However, no study to date has evaluated the content of the educational program or asked patients whether the content was pertinent to their needs [9]. Therefore, the understandability of the content of the offered patient education and the incentives that stimulate patients to “take action” and manage their own health are largely unexplored fields.

An educational intervention targeted at a health problem provides people with the opportunity to take a greater role in decisions on their health issues and to “take action” towards improving their quality of life. For this to function effectively, they must acquire the basic set of skills to be able to seek, understand and use health information. This is known as “health literacy”’. Health literacy is the degree to which individuals have the capacity to obtain, process and understand the basic health information and services needed to make appropriate health decisions [10, 11].

In a previous study, our team developed the Knee Osteoarthritis Patient Education Questionnaire (KOPEQ) to investigate the required content of a preoperative educational intervention from the patients’ perspective [9]. The development process was based on the conceptual framework of Wilson and Cleary [12, 13] and was guided by four health professionals from diverse backgrounds and two researchers. The KOPEQ comprised of; 1) 13 items to assess the content, using a formative model approach; and 2) three items to assess the clinical impact, adopting a reflective model approach. Likert items with a five-point scale were chosen as the scoring option. The feasibility, internal consistency and factor structure of this 16-item KOPEQ were investigated in a small cross-sectional study [9]. The internal consistency of the total scale was found to be 0.83 (95% CI 0.71–0.94). The exploratory factor analysis of the scale resulted in a 4-factor structure; subscales of didactics, addressability, empowerment and theory. Sixty-one percent of the variance was explained, while the loading of the separate items was between 0.47 and 0.96.

Based on the insights from the factor analysis and interviews with patients in this previous study, the following prerequisites for improving the construct validity of the 16-item KOPEQ were identified: a reduction in the number of items; further investigation of the construct validity of the measure; and, use of a larger sample size. In this follow-up study, our research team has further developed the KOPEQ based on these identified prerequisites.

Consequently, the main aims of this second study were to investigate the content validity including item reduction, construct validity and internal consistency of the 16-item KOPEQ. The participants, similarly, were patients with severe knee osteoarthritis who had routinely participated in a preoperative educational intervention prior to their knee replacement operation. The design used was a mixed method approach, integrating both quantitative and qualitative data collection. It included item reduction and hypotheses testing on validity.

Hypotheses on validity:

- Comparison of the constructs of the KOPEQ with a patient-testing interview protocol (IP).
  - Our primary hypothesis was that both measures are indicators of closely-related constructs and that a strong positive correlation [14] (above 0.7) will be found.
- Comparison of the constructs of the KOPEQ with the Short Test of Functional Health Literacy (S-TOFHLA) and the Mini-Mental State Examination (MMSE).
  - Our hypothesis was that the comparators for both constructs with the KOPEQ are only minimally related and that weak positive correlations (below 0.3) will be found. For the S-TOPHLA, the comparison between the KOPEQ and the following aspects were tested separately: a) reading comprehension; b) total time needed; and, c) number of points after 7 min.

## Methods

### Design

A mixed method design was used, integrating both quantitative and qualitative data collection. A focal point of this follow-up study was the evaluation of the content validity, including item reduction, of the KOPEQ. According to COSMIN guidelines, content validity covers the aspects of relevance, comprehensiveness and comprehensibility of a questionnaire. The steps of the process are described below in successive phases, using qualitative and quantitative methods within an integrated approach.

The study used a cross-sectional design and was embedded in the clinical practice of an orthopedic department of the cantonal hospital in Winterthur, Switzerland. Patients with severe knee OA, scheduled for a KR operation, were offered an educational intervention as part of the hospital’s routine care. This routine intervention was developed in our previous study [9]. The aim of the educational intervention is to impart practical knowledge to the patient through providing the following information: information and illustrative material on the anatomy of the knee and adjacent functional structures; recommendations on activities with a prothesis; information on postoperative pain management; and, details of the postoperative rehabilitation phase. Several authors recommend the use of targeted, easily understandable educational material to improve patients’ self-management, such as worksheets, handouts, presentations, photos and videos. The information provided must be prioritized according to importance, illustrated, conveyed in plain language and use short sentences without medical terms [15–19]. The educational intervention applied in this study consisted of two sessions (with one-week interval) and incorporated these recommendations.

### Population

Inclusion criteria were: proficiency in both spoken and written German: scheduled for KR surgery based on OA; under treatment at the cantonal hospital in Winterthur; and, a Mini-Mental State Examination score of greater than 24/30 [20]. During the first educational session, eligible participants were informed about the research project and, in addition, handed written documentation. One week later, following the second educational session, patients were asked whether they would agree to participate in the research study.

Those eligible patients agreeing to participate in the study completed the revised KOPEQ some six to 10 weeks after their KR surgery, having previously attended the two patient education sessions. During these same sessions, participants were also asked to complete the patient-testing (interview) protocol (IP) and to undergo the Short Test of Functional Health Literacy (S-TOFHLA) [21].

### Index measure

The intention of the KOPEQ is, from the patients’ perspective, to measure the understandability of a pre-operative educational intervention and to assess its actionability. Within the theoretical field of health literacy, patient education materials are described as understandable, “When consumers of diverse backgrounds (patients) and varying levels of health literacy can process and explain key messages” [22]. Patient education materials are described as actionable, “When consumers of diverse backgrounds and varying levels of health literacy can identify what they can do based on the information presented” [22].

The further development of the KOPEQ was executed in two phases in this study: 1) a reduction in the number of items on the initial 16-item KOPEQ; and 2) psychometric testing of internal consistency, factor analysis and construct validity. Reduction in the number of KOPEQ items was considered necessary because some placed too much focus on practical aspects (such as the number of intervention sessions). This was seen as, potentially, an impediment to the implementation of the KOPEQ in other contexts.

#### Phase 1: reduction in the number of items on the initial 16-item KOPEQ

We used the COSMIN methodology for assessing the content validity of patient-reported outcome measures (PROMs) as a methodological guide [23] in reducing the number of items. This guide includes ten criteria concerning the three aspects of content validity of relevance, comprehensiveness and comprehensibility. In this phase the number of items on the KOPEQ was reduced from 16 to 12.

The impact of the reduction of KOPEQ items was subsequently tested by an exploratory factor analysis (EFA).

#### Phase 2: hypotheses testing of the 12-item KOPEQ

The construct validity of the reduced KOPEQ was compared to an alternative measure with a comparable construct. An interview protocol (IP), tailored to the same patient educational material, was specifically developed for this study, according to comparator measures presented in a study by Shoemaker et al. [22]. The comparisons were based on a priori hypotheses regarding the strength and direction of the correlation of the constructs of the index and comparator measures. The primary correlation studied was between the sum score of the reduced KOPEQ to the sum score of the IP. The secondary correlations of interest were between the reduced KOPEQ and the Short Test of Functional Health Literacy (German version) and the Mini-Mental State Examination score.

#### Comparator measures

1. Interview protocol (IP)
  A patient-testing IP tailored to the same patient educational material [22], based on a mixed methods approach.

- Before the interview, patients are asked to randomly read or view selected material from the patient educational material
- The interviewer then asks a set of questions to investigate the patient’s understanding of the content of the material (understandability) and the extent to which they know what action to take (actionability). Patients could refer to the material as much as needed before answering the questions.
- There are 4 types of questions in the protocol:
  - Comprehension questions
  - Numeric-scoring questions (scale 1–10, how easy was the material to understand and/or to action)
  - Open-ended opinion questions
  - Questions that asked patients to describe what information was given in each session, or what a visual aid was showing

2. Short Test of Functional Health Literacy (S-TOFHLA)
  The S-TOFHLA [21] measures a patient’s ability to read and understand health-related materials. It consists of 40 items: 36 testing reading comprehension and four testing numeracy. Three aspects are measured:
  - Reading comprehension is assessed by a reading test using two health-related passages. Both passages have every 5th to 7th word deleted, and, for each blank word, the participant must select from a list of four words the ones that best complete the sentence. The numeracy test assesses the ability to read and understand numerical information in the form of prescription medication, appointment slips or other health-related material. The items are selected based upon their perceived importance and frequency of the task in the health-care setting.
  - The total time needed to complete the S-TOFHLA
  - The number of points after 7 min.

The Swiss, German-language validated version was used.

3. Mini-Mental State Examination (MMSE)
  The MMSE [20] is a widely-used test for screening cognitive functions. It includes tests of orientation, attention, memory, language and visual-spatial skills.

#### Potential confounder

It has been suggested that reading ability may deteriorate with age and have an influence on health literacy [24–28]. Therefore, age (measured in years) might be a confounder of the association between the KOPEQ and the IP.

### Sample size

For research questions regarding validity, a minimum number of 50 persons for an appropriate sample size is recommended [29]. In this study, we followed this recommendation in our choice of sample size. A formal sample size calculation was not performed.

### Phase 2 statistical analysis

- Comparison of the constructs of the reduced KOPEQ with the IP.
- Comparison of the constructs of the reduced KOPEQ with the S-TOPHLA and the MMSE.

Descriptive statistics were used to describe the population for all collected variables.

The internal consistency of the KOPEQ was investigated using Cronbach’s alpha [30] and a score above 0.70 was set as an indicator of sufficient reliability [14].

Exploratory factor analysis (EFA) was performed to assess the dimensionality of the KOPEQ.

To quantify the construct validity by testing the preset hypotheses, all pairwise correlations were estimated in an exploratory manner. Firstly, the KOPEQ sum score was correlated with the IP sum score. Secondly, the KOPEQ was correlated with the other comparators (S-TOFHLA 1–3 and MMSE). We computed Pearson’s correlation coefficient with bootstrapped 95% CI using Fisher transformation. Lastly, the correlations were adjusted for the potential confounder of age. In addition to the Pearson’s correlation, a Bayesian estimation of the main quantity of interest (the correlation between KOPEQ and IP) was performed by using uninformative priors. We used Gibbs to sample from the posterior distribution. A 95% Highest Posterior Density (HPD) interval for this correlation was constructed.

All analyses were performed using the R statistical software R version 3.5.2 (2018-12-20) [31]. For Bayesian analysis, we used JAGS (Just Another Gibbs Sampler).

A post hoc analysis, based on the qualitative information from the patient interviews, was optional for practicability reasons.

### Data storage and protection

Data was handled and stored confidentially on the ZHAW server and coded according to the rules of the Institute to protect the privacy of the participants. The principal investigator (PI) (EOH) kept the key of the code safeguarded. Only the PI had access to the code. The PI and two members of the project team (epidemiologist and statistician) had access to the data.

## Results

### Phase 1: reduction in the number of items on the initial 16-item KOPEQ

Through discussions within the expert team, the existing 16 items on the KOPEQ were reviewed against the aspect of relevance. This resulted in the removal of three items (“benefit of the imparted knowledge during hospitalization”, “benefit of the imparted knowledge after hospitalization” and “less fear of the time after surgery by the imparted knowledge”). The construct of these items did not conform directly to the defined content of the KOPEQ. A fourth item was removed because it was related to practical aspects of the intervention. For example, the preoperative patient education was offered in only two sessions in this follow-up study, compared with three sessions in the initial KOPEQ study.

Secondly, we reviewed the aspect of comprehensibility and changed the wording of two items, “How was the comprehensibility of the text in the handouts” and “How were my questions answered”, to make them more understandable.

Internal consistency analysis showed no notable change for the16 items versus the 12 items, with Cronbach’s Alpha of 0.83 (SE: 0.058) for the 16-item KOPEQ versus 0.88 (SE: 0.026) for the 12-item version.

Eigenvalue analysis and parallel analysis both yielded evidence for a two-factor model.

The remaining 12 items on the KOPEQ address more relevant issues, increase understandability and improve actionability for patients completing the educational intervention.

### Phase 2: hypotheses testing of the 12-item KOPEQ

Fifty percent of the eligible participants gave their written, informed consent. The remaining 50 % declined to participate in the (non-mandatory) educational sessions, giving their reasons as lack of time or lack of interest.

Of the total participating 50 patients, 35 were females and 15 males. Their education levels were distributed as follows: 1 person on level 1 (primary school); 3 persons on level 2 (secondary school); 36 persons on level 3 (apprenticeship); 1 person on level 4 (high/grammar school); 4 persons on level 5 (higher technical/business school); 2 persons on level 6 (university of applied sciences); and, 3 persons on level 7 (university or technical university).

The MMSE scores for all participants were above 24 points, resulting in no exclusions.

The Pearson’s correlation coefficient between: the 12-item KOPEQ and the IP was 0.72 (bootstrapped 95% CI: 0.47 to 0.88); between KOPEQ and S-TOPHLA was 0.10 (− 0.30 to 0.44), 0.00 (− 0.35 to 0.35) and 0.10 (− 0.30 to 0.44) respectively; and, between KOPEQ and MMSE was 0.10 (− 0.14 to 0.30). These correlations were not confounded by age (− 0.04 (− 0.30 to 0.27).

All five of our hypotheses were confirmed by the results.

### Phase 3: post hoc considerations and analyses

#### Second reduction of items (from 12-item to 7-item KOPEQ)

Following the main study analyses (phases 1 and 2), a post hoc analysis was undertaken. Based on qualitative information from the patient interviews, and for practicability reasons, another five items were removed from the KOPEQ: three items (“Division into three sessions”, “How was the session anatomy and function” and “How was the session recommended activities”) were not deemed content-relevant because they were concerned with organizational aspects; and two items (“How comprehensible was the imparted knowledge” and “How was the arrangement of the handouts”) because the content was already included in other items. A further reduction was not indicated since important information would then have been excluded. The following seven items were retained: Item 1, Item 3, Item 4, Item 5, Item 8, Item 11 and Item 12.

A synopsis of the reduction of the items from the initial 16-item KOPEQ to the 7-item KOPEQ is presented in Table 1.

**Table 1:**
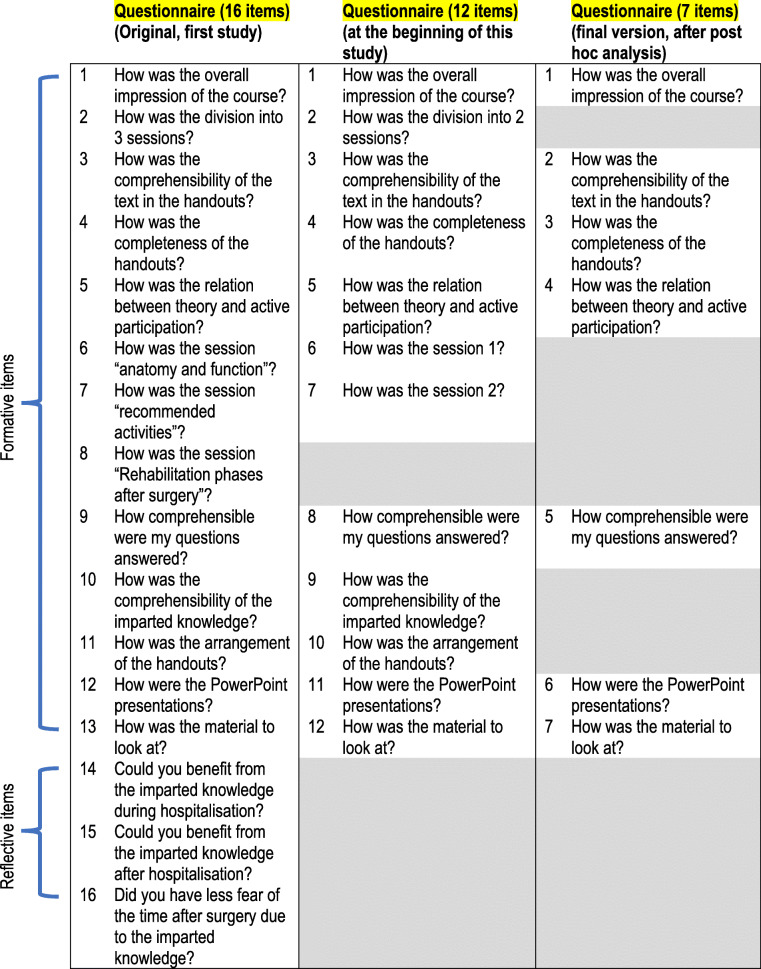
Synopsis of the reduction of the items: From 16 items towards 7 items

There were 50 complete observations on the 7-items of the final KOPEQ (sum score, mean (SD) = 91.9 (6.99)) and 31 complete observations of the IP items (sum score, mean (SD) = 88.9 (8.12)). The reading comprehension (S-TOPHLA) in points (mean (SD)) was 33.6 (1.89), the time needed in minutes was 10.6 (3.87) and the number of points after 7 min was 23.8 (7.61).

The characteristics of the variables are presented in Table 2.

**Table 2:** Characteristics of all variables

Items	Mean (SD)	Median	Min	Max
Age	72.4 (8.19)	73	53	89
MMSE	28.4 (1.48)	28.5	25	30
S-TOFHLA 1^a^	33.6 (1.89)	34.0	29	36
S-TOFHLA 2^a^	10.6 (3.87)	10.0	4.83	23.67
S-TOFHLA 3^a^	23.8 (7.61)	23.0	10	36
KOPEQ 1	90.5 (14.58)	95.0	15	100
KOPEQ 2	85.7 (18.77)	92.5	0	100
KOPEQ 3	91.8 (10.75)	95.0	52	100
KOPEQ 4	90.6 (11.18)	94.5	44	100
KOPEQ 5	91.8 (7.06)	92.5	72	100
KOPEQ 6	90.4 (11.6)	93.0	38	100
KOPEQ 7	89.5 (17.00)	94.5	0	100
KOPEQ 8	93.4 (6.74)	94.5	73	100
KOPEQ 9	92.7 (7.35)	95.0	71	100
KOPEQ 10	92.3 (7.97)	95.0	71	100
KOPEQ 11	92.7 (7.82)	95.0	73	100
KOEPQ 12	92.7 (7.95)	94.0	69	100
IP A1	91.4 (9.78)	94.5	55	100
IP A2	91.3 (8.60)	93.0	68	100
IP A3	92.1 (8.97)	94.0	62	100
IP A4	8.7 (15.41)	91.5	20	100
IP A5	91.8 (9.55)	95.0	64	100
IP A6	92.5 (7.50)	94.0	69	100
IP A7	92.4 (7.63)	93.5	66	100
IP A8	90.9 (14.82)	95.0	6	100
IP A9	92.0 (11.98)	96.0	48	100
IP A10	85.7 (15.26)	90.5	49	100
IP A11	94.5 (6.49)	98.0	73	100
IP A12	93.1 (10.10)	97.5	51	100
IP A13	76.9 (25.36)	81.0	0	100
IP A14	87.3 (15.99)	92.0	39	100
IP A15	93.7 (7.22)	96.0	73	100
IP A16	92.7 (8.41)	96.0	65	100
IP B1	92.9 (8.54)	96.0	60	100
IP B2	91.4 (12.61)	97.0	49	100
IP B3	71.5 (28.01)	81.5	0	100
IP B4	93.8 (7.22)	96.5	73	100
IP B5	94.0 (7.16)	96.0	74	100

^a^S-TOFHLA 1 (reading comprehension / 0–36 points), S-TOFHLA 2 (needed total time in minutes), S-TOFHLA 3 (number of points after 7 min)

Internal consistency analysis showed no notable change (12 items versus 7 items) with Cronbach’s Alpha of 0.88 (SE: 0.026) for the 12-item versus 0.84 (SE: 0.0357) for the 7-item version.

EFA analysis showed no evidence against a one-factor model with a ratio of 12.3 of the first (3.83) to the second eigenvalue (0.31).

#### Hypotheses testing of the 7-item KOPEQ

The Pearson’s correlation coefficient between; the 7-item KOPEQ and the IP was 0.77 (bootstrapped 95% CI: 0.60 to 0.89); between the 7-item KOPEQ and S-TOPHLA was 0.15 (− 0.18 to 0.44), − 0.02 (− 0.33 to 0.30) and 0.14 (− 0.24 to 0.46) respectively; and, between the 7-item KOPEQ and MMSE was 0.14 (− 0.12 to 0.14). These correlations were not confounded by age − 0.10 (− 0.37 to 0.15).

All pairwise correlations between the variables are presented in Table 3.

**Table 3:** Pearson’s correlation matrix of the KOPEQ with the other variables

Measure	1	2	3	4	5	6
1. KOPEQ	–					
2. IP	.77 (0.60 to 0.89)	–				
3. S-TOFHLA 1	.15 (−0.18 to 0.44)	−.23 (−0.57 to 0.18)	–			
4. S-TOFHLA 2	−.02 (−0.33 to 0.30)	.32 (0.07 to 0.55)	−.43 (−0.63 to −0.16)	–		
5. S-TOFHLA 3	.14 (− 0.24 to 0.46)	−.23 (− 0.57 to 0.11)	.59 (0.40 to 0.72)	−.87 (− 0.94 to − 0.78)	–	
6. MMSE	.14 (−0.12 to 0.14)	.05 (− 0.28 to 0.34)	.13 (− 0.13 to 0.39)	−.11 (− 0.37 to 0.11)	.20 (− 0.07 to 0.49)	–

Values are presented as Pearson’s correlation coefficient with 95% Confidence Interval

*KOPEQ* (Knee Osteoarthritis Patient Education Questionnaire), *IP* (Interview Protocol), *S-TOFHLA* (Short Test of Functional Health Literacy; 1: reading comprehension; 2: total time needed; 3: number of points after 7 min), *MMSE* (Mini-Mental State Examination)

**Fig. 1:**
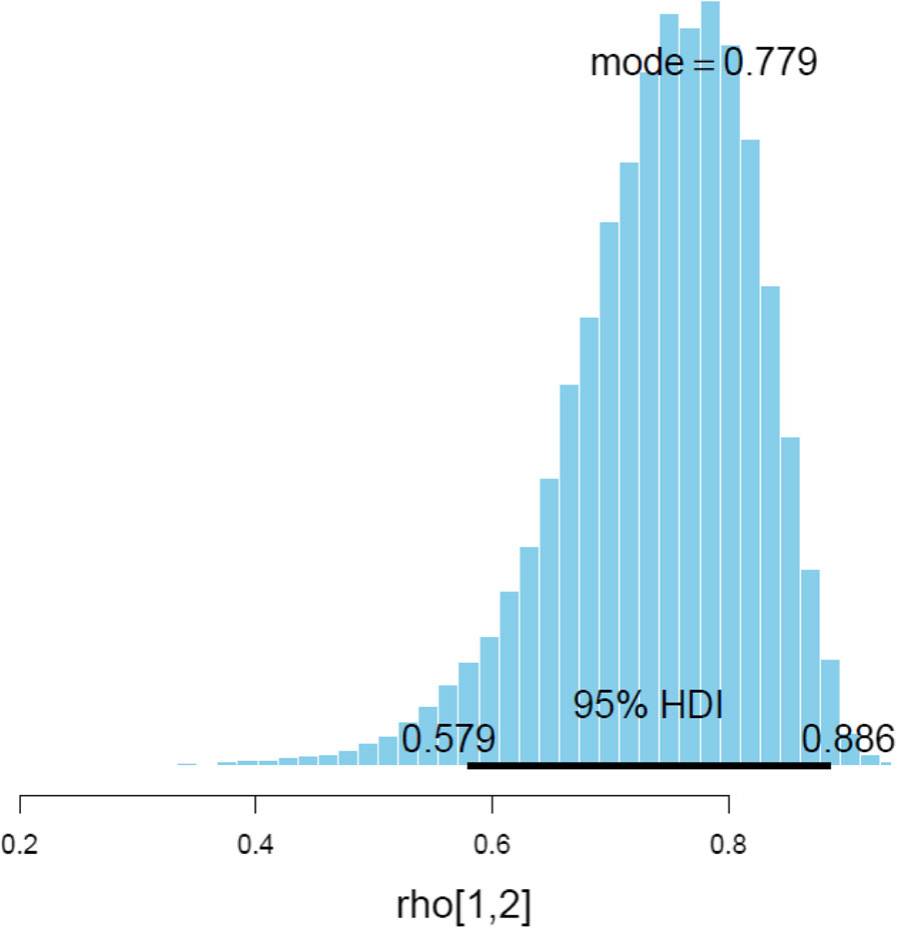
Posterior Distribution of the correlation of the KOPEQ versus IP. HDI (High Density Interval). KOPEQ (Knee Osteoarthritis Patient Education Questionnaire) IP (Interview Protocol)

The Bayesian posterior distribution of the correlation between the 7-item KOPEQ and the IP is shown in Fig. 1 (mean: 0.74, mode: 0.78, 95% HPD: 0.58 to 0.89).

All five of our hypotheses were confirmed (Table 3).

The original German version, as well as an English version, of the 7-item patient education questionnaire, are now available for clinicians (see Additional files 1 and 2).

## Discussion

This article describes a second study on the validation of our initial 16-item KOPEQ [9]. During this study, we realized that the title of the questionnaire KOPEQ is not the optimal name for the questionnaire. To more accurately reflect the content of the included items following further validation, the title of the 7-item Knee Replacement Patient Education Questionnaire was changed to KR-PEQ-7.

The construct validity was tested by comparing the KR-PEQ 7 (former title “KOPEQ”) with related measures based on a priori hypotheses. All five hypotheses were confirmed. The KR-PEQ-7 provides short, clinically useful measures on the understandability and comprehensiveness of a patient education intervention prior to a knee replacement operation.

### Removing five items

A questionnaire should be as short as scientifically possible because it increases its feasibility and practicability, which is important for use in clinical practice. The content and statistically driven reduction from 12 to 7 items was justifiable: removal of items 2, 6 and 7 is based on the argument that these items addressed organizational issues and not content. How to best organize a patient education intervention is controversial. The educational format can vary significantly, from the simple delivery of an information booklet or video, to face-to-face verbal communication, to multiple group sessions [32–36]. It is therefore justified, in order to improve the feasibility and practicability of the questionnaire, to remove all items concerning the aspect of organization. Removal of item 9 was the outcome of an investigation into internal consistency and was based on its high correlation with items 11 and 12, thus providing no additional information. Item 10 was removed because it was already included in items 3 and 4. Generally, we assume that these removals are in line with the COSMIN recommendations [14]. The 7-item KR-PEQ-7 shows strong internal consistency.

### Construct validity by hypotheses testing

The primary a priori hypothesis on the association between the construct of the KR-PEQ-7 and the IP (positive correlation above 0.7) was confirmed with a Pearson’s correlation coefficient of 0.77 (95% CI 0.60–0.89).

Our four secondaries a priori hypotheses were also confirmed, with Pearson’s correlation coefficients below 0.30.

### Health literacy level of the study population

The mean time that participants took to complete the S-TOFHLA in our study was 10.58 (3.87) minutes, which accords with the completion time of 12 min or less for the original S-TOFHLA [37]. In the German version of the S-TOFHLA, the test must be stopped after 7 min, and the cut-off graded. “Adequate health literacy” at this cut-off is deemed to be 23 points [21]. Our patients achieved a mean value of 33.6 (1.89) points with no time limitation and a mean value of 23.8 (7.61) points at the seven-minute cut-off. This is at the lower end, meaning that some of the patients did not achieve the grade of adequate health literacy. However, with no the time limitation, our population showed good health literacy. In addition, the MMSE scores for all patients were above 24 points. Hence, we assume that the study population was competent to complete the questionnaire and respond to the questions of the IP measure.

Age did not influence our questionnaires and older people were shown to be capable of completing them. This is in line with the results for the S-TOFHLA [21], although several studies have reported that levels of health literacy decrease with increasing age [24–28]. Our result may be explained by the fact that older people with health conditions are likely to have accumulated a significant amount of knowledge about their condition over a lengthy period of time, which has been shown to increase health literacy levels [38].

### Limitations

Our study has limitations. We only used a sample of KR patients recruited from the cantonal hospital in Winterthur and only 50% of the eligible people participated. This limits the generalizability of the results.

## Conclusions

The KR-PEQ-7 (former KOPEQ) shows good psychometric properties and provides short, clinically useful measures for the understandability and comprehensiveness of a patient education intervention that takes place prior to a knee replacement operation.

Clinicians could use the KR-PEQ-7 to receive valid feedback on how patients with OA on a waiting list for KR judge the applied patient education intervention.

Further investigation is needed to assess the applicability of the KR-PEQ-7 in larger samples in different hospitals and countries and to investigate other psychometric properties, such as test-retest reliability.

Additionally, further research is encouraged to validate the 7-item Patient Education Questionnaire in a population other than knee replacement patients.

## Supplementary information

**Additional file 1.** KR-PEQ-7: German version, original

**Additional file 2.** KR-PEQ-7: English version, not cross-culturally validated

## Data Availability

The datasets collected during and/or analyzed during the current study are available from the corresponding author on reasonable request.

## Abbreviations

COSMIN: (COnsensus-based Standards for the selection of health Measurement INstruments)
IP: Interview protocol
KOPEQ: Knee osteoarthritis patient education questionnaire
KR-PEQ-7: 7-Item knee replacement patient education questionnaire
KR: Knee replacement
MMSE: Mini-mental state examination
OA: Osteoarthritis
PI: Principal investigator
PBT: Performance-based test
PROMs: Patient-reported outcome measures
S-TOFHLA: Short test of functional health literacy
