# Supplementary material for: Validation of the 7-item knee replacement patient education questionnaire (KR-PEQ-7), based on the 16-item knee osteoarthritis patient education questionnaire (KOPEQ)
Source: BMC Musculoskelet Disord. 2020 Jul 16;21:468. doi: 10.1186/s12891-020-03476-y (PMC7367315; doi:10.1186/s12891-020-03476-y)
Supplement: Supplementary file 1 — Additional file 1. KR-PEQ-7: German version, original [file 12891_2020_3476_MOESM1_ESM.docx]

**KR-PEQ-7**(Knee Replacement - Patient Education Questionnaire – 7 Fragen)

Name Patient/in: ____________________________ Datum: ____________________

Sie haben vor Ihrer Knieoperation 2 Schulungen besucht. Uns interessiert was Ihnen gefallen oder auch missfallen hat. Ihre Eindrücke sind wichtig, damit wir die Qualität der Schulungen verbessern können.

Beantworten Sie bitte jede Frage durch ankreuzen des zugehörigen Kästchens. Bitte nur ein Kästchen pro Frage ankreuzen. Wenn Sie sich unsicher sind, wie Sie die Frage beantworten sollen, wählen Sie die Antwort aus, die Ihnen am zutreffendsten erscheint.

|  | | **Sehr gut** | **Gut** | **Genügend** | **Ungenügend** | **Schlecht** |
| --- | --- | --- | --- | --- | --- | --- |
|  | | **5** | **4** | **3** | **2** | **1** |
| 1 | Wie war ihr Gesamteindruck der Schulung? | **□** | **□** | **□** | **□** | **□** |
| 2 | Wie empfanden sie die Verständlichkeit der Texte in den Kursunterlagen? | **□** | **□** | **□** | **□** | **□** |
| 3 | Wie empfanden sie die Vollständigkeit der Kursunterlagen? | **□** | **□** | **□** | **□** | **□** |
| 4 | Wie empfanden sie das Verhältnis zwischen Theorie und aktiver Mitarbeit? | **□** | **□** | **□** | **□** | **□** |
| 5 | Wie verständlich wurden ihre Fragen beantwortet? | **□** | **□** | **□** | **□** | **□** |
| 6 | Wie fanden sie die Leinwandpräsentationen? | **□** | **□** | **□** | **□** | **□** |
| 7 | Wie fanden sie das praktische Anschauungsmaterial? | **□** | **□** | **□** | **□** | **□** |

Total Punkte : _________ Mittelwert (Total : 7) : _________
