# Supplementary material for: Validation of the 7-item knee replacement patient education questionnaire (KR-PEQ-7), based on the 16-item knee osteoarthritis patient education questionnaire (KOPEQ)
Source: BMC Musculoskelet Disord. 2020 Jul 16;21:468. doi: 10.1186/s12891-020-03476-y (PMC7367315; doi:10.1186/s12891-020-03476-y)
Supplement: Supplementary file 2 — Additional file 2. KR-PEQ-7: English version, not cross-culturally validated [file 12891_2020_3476_MOESM2_ESM.docx]

**KR-PEQ-7**(**K**nee **R**eplacement - **P**atient **E**ducation **Q**uestionnaire - **7** questions)

Name of patient: ____________________________ Date: ____________________

Before your knee operation, you attended two introductory courses. We are interested to hear about what you liked or disliked in these courses. Your impressions are important to help us improve the quality of our program.

Please answer all questions by ticking the box that represents your experience most closely. Only one box per question should be ticked. When you are unsure how to answer the question, you should choose the answer that seems the most appropriate.

|  | | **Very good** | **Good** | **Satisfactory** | **Unsatisfactory** | **Poor** |
| --- | --- | --- | --- | --- | --- | --- |
|  | | **5** | **4** | **3** | **2** | **1** |
| 1 | How was the overall impression of the course? | **□** | **□** | **□** | **□** | **□** |
| 2 | How was the comprehensibility of the text in the handouts? | **□** | **□** | **□** | **□** | **□** |
| 3 | How was the completeness of the handouts? | **□** | **□** | **□** | **□** | **□** |
| 4 | How was the relation between theory and active participation? | **□** | **□** | **□** | **□** | **□** |
| 5 | How comprehensible were my questions answered? | **□** | **□** | **□** | **□** | **□** |
| 6 | How were the PowerPoint presentations? | **□** | **□** | **□** | **□** | **□** |
| 7 | How was the material to look at? | **□** | **□** | **□** | **□** | **□** |

Total Points: _________ Average (Total: 7): _________
